# Beyond the scalpel: forensic patterns, epistemic limits, and the medico-legal classification of femicide

**DOI:** 10.1016/j.clinsp.2026.101144

**Published:** 2026-09-09

**Authors:** Carmen Silvia Molleis Galego Miziara, Ivan Dieb Miziara

**Affiliations:** Department of Legal Medicine, Bioethics, Occupational Health and Physical Medicine and Rehabilitation, Faculdade de Medicina da Universidade de São Paulo (FMUSP), São Paulo, SP, Brazil

**Keywords:** Femicide, Forensic medicine, Autopsy, Gender-based violence, Homicide, Expert testimony

## Abstract

**Objective:**

To synthesize forensic autopsy findings reported in femicide and to evaluate their contribution, limitations, and appropriate use in medico-legal classification.

**Methods:**

A structured narrative review was conducted in PubMed/MEDLINE, SciELO, and ScienceDirect for publications from January 2000 to March 2026. Search terms addressed femicide, gender-related homicide, forensic pathology, autopsy, injury patterns, and forensic evidence. Sources providing forensic, autopsy, injury-pattern, or analytical medico-legal data were included in the main synthesis. Epidemiological and public health studies were retained only for contextual interpretation.

**Results:**

Ten forensic or analytical sources were retained for the main narrative synthesis, and ten epidemiological or public health sources were retained for contextual framing. Recurrent findings included overkill, craniofacial and cervical injuries, asphyxial mechanisms, defensive wounds, and weapon-specific patterns. However, these findings were non-specific and did not reliably distinguish intimate partner femicide from non-intimate partner femicide. The Belgrade autopsy series reinforced the importance of domestic setting and firearm use while showing substantial overlap in injury morphology between femicide subtypes.

**Conclusion:**

Femicide is associated with a recognizable but non-specific forensic profile. Morphology alone cannot establish gender-related motivation or legal classification. Expert interpretation should integrate autopsy findings with crime-scene reconstruction, victim-perpetrator relationship, prior violence, weapon access, and sociobehavioral context. Standardized documentation and integrated medico-legal frameworks may improve forensic consistency, judicial communication, and public health surveillance.

## Highlights

• Femicide shows recurrent but non-specific forensic patterns.
• Overkill, craniofacial injuries, and asphyxia are frequent.
• Injury patterns do not reliably differentiate IPF from NIPF.
• Morphology alone cannot establish gender-related motivation.
• Integrated forensic and contextual analysis is essential.

## Introduction

Femicide is the intentional killing of women or girls in which gender-related factors are part of the motivational, relational, or contextual structure of the death. It represents the most severe endpoint of violence against women and lies at the intersection of public health, criminology, human rights, and forensic medicine.^1^, ^2^, ^3^, ^4^, ^5^ Unlike generic homicide, femicide is not defined exclusively by the biological sex of the victim or by the immediate mechanism of death. It requires consideration of gender inequality, power asymmetry, coercive control, prior abuse, misogyny, sexual violence, and other circumstances that may indicate gender-related motivation.^3^, ^4^, ^5^

Global evidence shows that violence against women is highly prevalent and frequently perpetrated by intimate partners. Physical or sexual intimate partner violence affects a substantial proportion of women worldwide, and intimate partner homicide accounts for a major share of female homicides.^1^^,^^2^^,^^5^^,^^19^ Femicide should therefore be understood as the lethal endpoint of a continuum that may include recurrent abuse, threats, stalking, separation conflict, strangulation, coercive control, and escalation of violence.^4^^,^^5^^,^^19^

Forensic medicine has a central but delimited role in this field. Autopsy evidence may identify the cause and mechanism of death, injury distribution, vitality of lesions, defensive wounds, sexual violence markers, toxicological factors, and the degree of violence applied to the body. These data may support reconstruction and may indicate interpersonal proximity, excessive violence, restraint, resistance, or concealment. However, the autopsy does not, by itself, establish motive. Gender-related motivation is an inferential construct that depends on convergence between anatomical, investigative, relational, and sociobehavioral evidence.^4^^,^^7^, ^8^, ^9^, ^10^, ^11^, ^14^, ^15^

The forensic literature repeatedly describes overkill, craniofacial trauma, cervical injuries, strangulation, multiple mechanisms, and defensive lesions in cases classified as femicide.^7^, ^8^, ^9^, ^10^, ^11^ These findings are important because they may indicate proximity, escalation, humiliation, domination, or symbolic aggression. Nevertheless, their specificity is limited. Similar injury patterns may occur in non-gendered interpersonal homicides, emotionally driven homicides, robbery-related deaths, or other lethal events involving close contact.^8^^,^^9^^,^^12^

Recent autopsy-based evidence from Belgrade is particularly relevant because it directly compared intimate partner femicide and non-intimate partner femicide. That series reported a predominance of intimate partner femicide, frequent occurrence in domestic settings, and high firearm use, but did not identify injury patterns that reliably differentiated intimate partner from non-intimate partner femicide.^18^ This finding reinforces a core medico-legal problem: the motivational structure of femicide may not be visible as a distinct anatomical pattern.

This narrative review aims to synthesize current forensic and epidemiological evidence on femicide and to evaluate the extent to which autopsy findings contribute to medico-legal characterization. The specific objectives are: (i) To identify recurrent forensic patterns described in femicide; (ii) To assess whether these patterns can distinguish intimate partner femicide from non-intimate partner femicide; (iii) To examine the epistemic limits of morphology in establishing gender-related motivation; and (iv) To propose an integrated medico-legal framework for forensic reporting and legal communication.

## Methods

### Design

This study was designed as a structured narrative review. A narrative approach was chosen because the available evidence is heterogeneous with respect to definitions of femicide, legal classification, autopsy protocols, data sources, and reporting standards. The objective was not to estimate pooled prevalence or diagnostic accuracy, but to provide a critical medico-legal synthesis of recurrent forensic findings and their interpretative limits.

### Search strategy

A literature search was conducted in PubMed/MEDLINE, SciELO, and ScienceDirect for publications from January 2000 to March 2026. Search terms were applied to title, abstract, and keyword fields whenever database functionality allowed field specification; when this was not available, the closest equivalent broad search field was used. The following search strategy was adapted to each database: ("femicide" OR "gender-related homicide" OR "intimate partner homicide" OR "lethal violence against women") AND ("forensic pathology" OR "autopsy" OR "injury patterns" OR "mechanism of death" OR "forensic evidence" OR "medico-legal").

No geographic restriction was applied. No formal language restriction was applied at the search stage; however, full interpretation was limited to studies available in English, Portuguese, Spanish, or with sufficient English-language data in the abstract or tables. This limitation was retained because accurate interpretation of forensic variables requires reliable access to methods and results.

### Eligibility criteria

Sources were eligible for the main forensic synthesis when they provided primary forensic, autopsy, injury-pattern, or analytical data relevant to femicide, intimate partner homicide, gender-related homicide, or lethal violence against women. Eligible variables included mechanism of death, weapon type, anatomical distribution of injuries, overkill, defensive wounds, sexual violence indicators, victim-perpetrator relationship, scene context, or medico-legal interpretation.

Sources were excluded from the main forensic synthesis when they consisted solely of theoretical discussion, legal commentary without empirical or forensic data, studies of non-lethal violence only, or reports with insufficient methodological clarity. Epidemiological and public health studies were retained only when they contributed to contextual interpretation of femicide, intimate partner homicide, risk factors, surveillance, or underreporting.

### Selection and extraction

Titles and abstracts were screened by two reviewers. Full texts were assessed for eligibility. Disagreements were resolved by consensus. Extracted variables included study design, setting, sample size when available, population or data source, mechanism of death, weapon or method, injury distribution, defensive injuries, overkill, victim-perpetrator relationship, scene setting, and medico-legal contribution.

### Scope of synthesis and quality appraisal

Because this is a narrative review, no formal risk-of-bias or quality assessment tool was applied. This is acknowledged as a limitation. The synthesis separated two evidence layers: (i) Forensic/autopsy and analytical sources, summarized in Table 1; and (ii) Epidemiological and public health sources, summarized in Table 2 and used only for contextual framing. This separation was adopted to avoid conflating primary morphological evidence with population-level or conceptual evidence.

**Table 1:** Forensic and analytical sources included in the narrative synthesis of femicide: characteristics and medico-legal contribution.

Author / Year	Setting	Design / Source type	n	Key forensic findings	Medico-legal contribution
Di Nunno et al. (2019)	Italy / Forensic literature	Systematic forensic review	> 50 cases reviewed	Multiple injuries, overkill, combined trauma	Supports interpretation of escalation and multimodal aggression.
Cecchi et al. (2015)	Italy	Autopsy / forensic pathology series	30	Craniofacial injuries; blunt and sharp trauma	Suggests proximity and possible anatomical targeting.
Zara and Gino (2022)	Multicenter / Criminological-forensic	Analytical review	Not applicable	Overkill in intimate contexts	Links injury excess to relational dynamics but not specificity.
Santoro et al. (2014)	Italy	Medico-legal analysis	Not reported	Overkill patterns	Clarifies interpretative value and limits of overkill.
Byard (2019)	Forensic pathology literature	Diagnostic review	Not applicable	Strangulation; subtle or underdiagnosed findings	Highlights need for standardized neck examination.
Liem et al. (2011)	International	Comparative homicide analysis	Not applicable	Patterns of lethal interpersonal violence	Provides comparative context beyond femicide.
Palladino et al. (2011)	United States	Epidemiological / forensic context	Not specified here	Homicide during pregnancy/postpartum	Expands vulnerable-population considerations.
Maguire et al. (2009)	United Kingdom	Clinical-forensic comparative evidence	Not femicide-specific	Cranial injury interpretation	Included only as comparative support for trauma interpretation.
Dirkmaat et al. (2008)	United States	Forensic anthropology review	Not applicable	Skeletal trauma; perimortem injury	Relevant in decomposed or skeletonized remains.
Belgrade Femicide Study (2026)	Serbia	Retrospective autopsy series	53	Firearm use; domestic setting; no reliable IPF-NIPF injury differentiation	Shows overlap in morphology despite distinct relational contexts.

IPF, Intimate Partner Femicide; NIPF, Non-Intimate Partner Femicide. Entries listed as not femicide-specific were retained only as methodological or comparative support and not as primary evidence of femicide-specific morphology.

**Table 2:** Epidemiological and public health sources used for contextual framing.

Source / Year	Evidence type	Contextual contribution to the review
Sardinha et al. (2022)	Global prevalence estimates	Provides population-level context for intimate partner violence against women.
Garcia-Moreno et al. (2015)	Health-system / Public health review	Frames violence against women as a health-system and institutional response issue.
UNODC (2019)	Global homicide surveillance	Defines gender-related killing within international crime and surveillance frameworks.
Campbell et al. (2003)	Case-control femicide risk study	Identifies prior abuse, threats, separation, and firearm access as risk factors.
Stoeckl et al. (2013)	Systematic review	Estimates global prevalence of intimate partner homicide.
Vives-Cases et al. (2016)	Public health perspective	Discusses femicide and gender-based violence as epidemiological phenomena.
Abrahams et al. (2014)	Systematic review	Provides broader context of non-partner sexual violence.
Krug et al. (2002)	WHO violence framework	Situates femicide within interpersonal and collective violence prevention.
Devries et al. (2013)	Global burden evidence	Supports the health burden and prevention relevance of intimate partner violence.
Sumner et al. (2015)	Violence prevention perspective	Supports surveillance, prevention, and institutional response considerations.

**Fig. 1:**
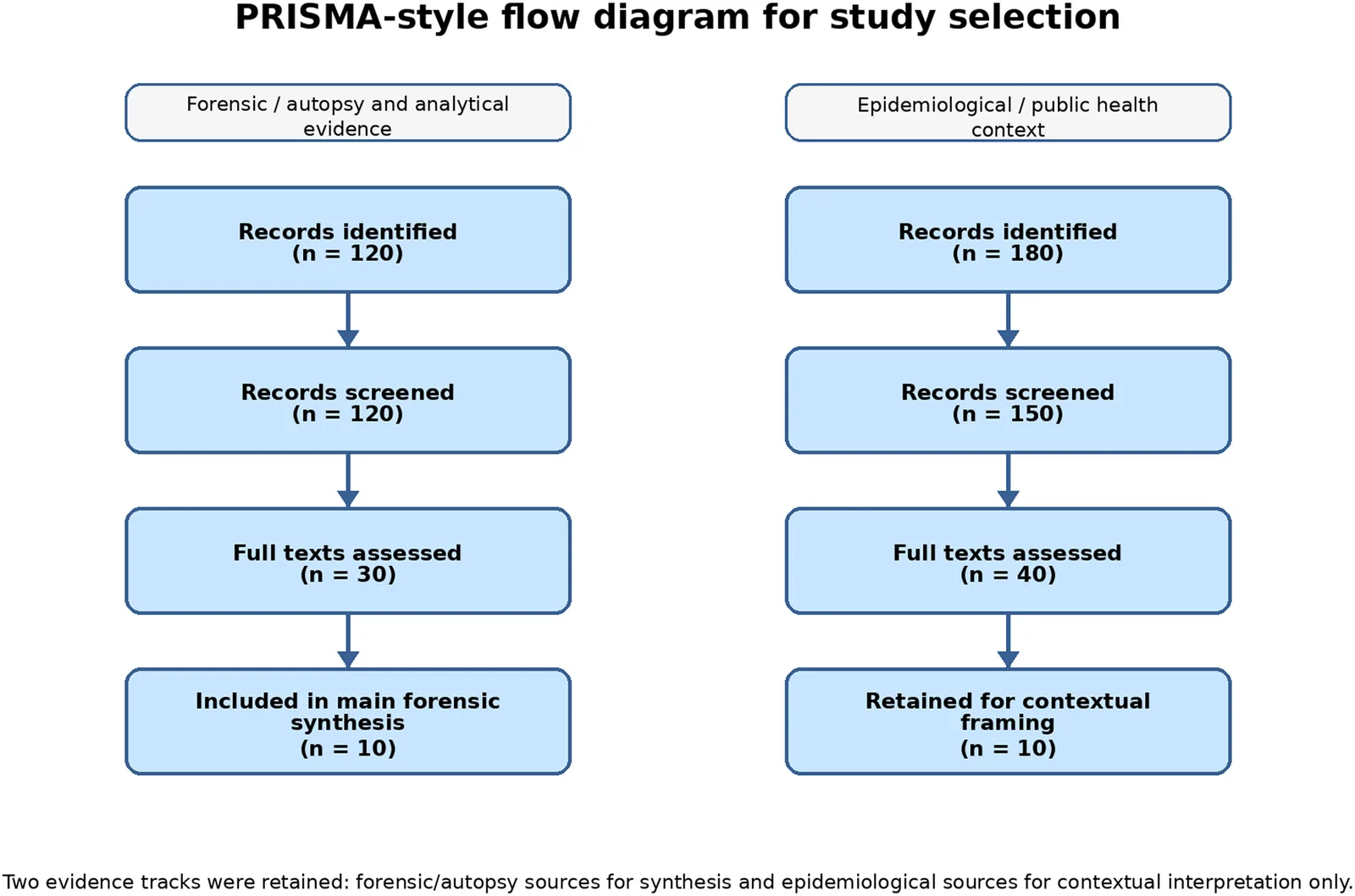
PRISMA-style flow diagram for study selection. Ten forensic/autopsy or analytical sources were included in the main synthesis; ten epidemiological or public health sources were retained for contextual framing only.

The study selection process is presented in Fig. 1. The figure is included in the main manuscript, rather than as supplementary material, to avoid ambiguity regarding the evidence tracks used in the review.

## Results

### Study selection and evidence structure

The final synthesis retained ten forensic or analytical sources for the main medico-legal review and ten epidemiological or public health sources for contextual framing. The two evidence groups were intentionally separated. Forensic sources inform the interpretation of mechanisms of death and injury morphology. Epidemiological sources inform the broader context of intimate partner violence, surveillance, risk factors, underreporting, and public health implications.

### Forensic/autopsy findings

Across the forensic literature, femicide was associated with recurrent but non-exclusive patterns. The most consistently described findings were: multiple injuries or overkill; craniofacial, cervical, or upper-body trauma; asphyxial mechanisms, especially strangulation; defensive wounds; and injuries compatible with close-contact assault. These findings contribute to reconstruction but do not establish gender-related motivation in isolation.

Overkill was the most conceptually prominent pattern. It may reflect emotional intensity, rage, domination, or escalation in intimate contexts. However, overkill is an interpretative descriptor rather than a diagnostic criterion. It requires operational definition, such as injury number exceeding what is necessary to cause death, and must be interpreted with scene and relational evidence.

Craniofacial and cervical injuries were also repeatedly reported. Facial injury may reflect proximity, interpersonal confrontation, depersonalization, or symbolic targeting. Neck trauma and strangulation are particularly important because they may reflect control, domination, and prior non-lethal strangulation. Nevertheless, detection of strangulation is strongly dependent on autopsy protocol and neck dissection technique.

Defensive wounds provide evidence of active resistance or struggle, usually on the hands, forearms, and upper limbs. Their absence does not exclude femicide because victims may be incapacitated, asleep, restrained, intoxicated, surprised, elderly, disabled, or attacked with firearms. Therefore, defensive injuries should be documented but not used as a binary criterion.

### Belgrade autopsy series and firearm use

The Belgrade series added contemporary autopsy-based evidence by showing that intimate partner femicide accounted for most cases, domestic environments were frequent, and firearms represented a prominent mechanism of death.^18^ This is important because several classic forensic discussions emphasize overkill, sharp-force injury, blunt trauma, or strangulation, whereas firearm prevalence may substantially alter the visible injury profile.

The same study did not find injury patterns that reliably differentiated intimate partner femicide from non-intimate partner femicide.^18^ This result is medico-legally important. It indicates that the motivational structure of the crime and the morphological structure of the body do not necessarily map onto each other.

### Geographic, temporal, and vulnerable-population considerations

Most forensic studies available for synthesis were concentrated in European or high-income settings, with limited representation from Latin America, Africa, Asia, and other regions where femicide definitions, weapon availability, reporting systems, and legal categories may differ. Consequently, generalizability remains limited. Temporal changes in firearm access, domestic violence legislation, forensic awareness, and surveillance may also influence the injury profiles reported over time.

Elderly femicide deserves particular attention. Older women may present fewer defensive wounds because of frailty, comorbidities, reduced mobility, cognitive impairment, medication use, dependency, or surprise attacks. In these cases, femicide may overlap with neglect, caregiver violence, financial exploitation, or misclassified accidental and natural deaths.

### Contextual epidemiological evidence

Epidemiological studies support the interpretation of femicide as the lethal endpoint of a broader continuum of violence against women. These sources identify intimate partner violence, prior abuse, coercive control, separation, stalking, firearm access, pregnancy or postpartum vulnerability, and structural inequalities as relevant contextual factors. They were not treated as primary autopsy evidence but as essential background for medico-legal interpretation.

## Discussion

### Main interpretation

The central finding of this review is that femicide has a recognizable but non-specific forensic profile. Overkill, craniofacial trauma, cervical injuries, asphyxial mechanisms, defensive wounds, and multiple mechanisms are repeatedly described in femicide and intimate partner homicide.^7^, ^8^, ^9^, ^10^, ^11^^,^^18^ However, none of these findings is pathognomonic. They can support reconstruction, suggest interpersonal proximity, and raise suspicion of gender-related violence, but they cannot independently establish femicide or differentiate intimate partner femicide from non-intimate partner femicide.^4^^,^^5^^,^^8^^,^^18^

This distinction is crucial for forensic practice. A forensic expert may state that findings are compatible with close-range interpersonal violence, prolonged assault, struggle, restraint, or repeated force. The expert should not state that femicide is established solely because overkill, facial injury, strangulation, or defensive wounds are present. Gender-related motivation depends on convergent evidence that extends beyond the body.^3^, ^4^, ^5^^,^^17^^,^^20^

### Epistemic limits of morphology

The term “epistemic limits” refers to the boundary of what can be known from the body alone. Autopsy findings identify how death occurred and may indicate dynamics of assault. They do not, by themselves, prove why the killing occurred. Motivation is inferred from convergent evidence: relationship history, prior threats, domestic violence records, stalking, coercive control, separation conflict, sexual violence, misogynistic behavior, witness statements, and scene investigation.^4^^,^^5^^,^^17^^,^^19^

The divergence between motivational structure and injury morphology explains why intimate partner femicide and non-intimate partner femicide may be difficult to differentiate anatomically. The same weapon, anatomical target, or mechanism can appear in different relational contexts. Conversely, similar motivational structures can result in different mechanisms of death depending on weapon access, opportunity, setting, and victim vulnerability.^8^^,^^12^^,^^18^

### Forensic patterns: contribution and limitations

Overkill is frequently discussed as a possible indicator of relational violence, emotional intensity, domination, or symbolic aggression.^7^, ^8^, ^9^ Nevertheless, it remains a probabilistic and context-dependent descriptor. Overkill may occur in intimate partner femicide, but it may also occur in other interpersonal homicides. Its evidentiary value depends on operational definition and on correlation with scene, relational, and behavioral evidence.^8^^,^^9^^,^^12^

Craniofacial injuries may be relevant because the face is anatomically exposed and symbolically associated with identity. Such injuries may suggest proximity, confrontation, humiliation, or depersonalization.^7^^,^^10^ However, they can also reflect the mechanics of assault, such as frontal confrontation, blunt trauma, falls after impact, or repeated blows during struggle. Therefore, reports should describe injury number, distribution, depth, direction, and associated lesions before advancing interpretative hypotheses.^7^^,^^10^

### Defensive wounds and resistance

Defensive wounds are useful but context-dependent markers. Incised wounds on the palms and fingers, blunt injuries on the forearms, and patterned injuries on the upper limbs may support a struggle. Their distribution can help reconstruct relative positions, weapon type, and victim awareness. However, the absence of defensive wounds does not exclude femicide. Victims may be asleep, intoxicated, incapacitated, restrained, surprised, elderly, disabled, or killed rapidly by firearms.^10^^,^^18^

In intimate partner femicide, a lack of defensive wounds may also reflect coercive control, fear, unequal physical strength, dependency, or attack in a private domestic setting. Thus, defensive injuries should be documented systematically but interpreted cautiously. They are evidence of resistance when present, not evidence against femicide when absent.^4^^,^^5^^,^^10^

### Firearms and setting-specific morphology

Firearm use changes the forensic profile of femicide. It may result in fewer external injuries, fewer defensive wounds, and a shorter assault interval. It also expands the relevance of ballistics, range-of-fire assessment, trajectory analysis, gunshot residue, weapon ownership, licensing, access by the perpetrator, and scene reconstruction. The Belgrade autopsy series is important because it showed high firearm use and frequent domestic settings, while also demonstrating that injury patterns did not reliably separate intimate partner femicide from non-intimate partner femicide.^18^

The prevalence of firearms is context dependent. In settings with high firearm access, femicide may appear as a single gunshot wound rather than as overkill, strangulation, or multiple sharp-force injuries.^18^ In settings with lower firearm access, other mechanisms may predominate. This observation reinforces that mechanism of death is shaped by environment and opportunity, not only by motive.^3^^,^^12^^,^^18^

### Geographic variability, temporal change, and generalizability

The generalizability of the forensic literature remains limited. Available studies are concentrated in selected European or high-income settings, while regions with high femicide burdens, different weapon availability, and variable forensic infrastructure remain underrepresented.^3^^,^^6^^,^^16^^,^^17^^,^^20^ Findings derived from Italian or European series should therefore not be extrapolated without caution to Latin America, Africa, Asia, or North America.

Temporal changes may also influence femicide morphology. Changes in firearm availability, domestic violence legislation, reporting practices, emergency response, forensic training, and social recognition of coercive control can alter which cases are detected and how they are classified. Current evidence does not permit firm conclusions on temporal trends, but future studies should examine whether patterns of overkill, strangulation, firearm use, defensive wounds, and domestic setting change across time and jurisdictions.^10^^,^^18^

### Elderly femicide and vulnerable victims

Elderly femicide is insufficiently examined in the forensic literature. Older women may be more vulnerable because of frailty, disability, cognitive impairment, social isolation, dependency, caregiving asymmetry, or reduced capacity for physical resistance. Their deaths may present with fewer defensive injuries, lower-energy mechanisms, neglect, smothering, poisoning, blunt trauma, or findings initially interpreted as accidental or natural.^10^^,^^17^

Age-sensitive assessment should therefore document dependency, caregiving relationships, prior neglect, financial exploitation, medication exposure, cognitive impairment, comorbidities, and scene circumstances. The absence of classic femicide patterns should not lead to exclusion of gender-related or relational violence in older victims.^4^^,^^10^^,^^17^

### Epidemiological evidence as contextual support

Epidemiological evidence incorporated into this review was used for contextual framing, not as primary morphological evidence. Global studies show that violence against women and intimate partner violence are prevalent and that intimate partner homicide is a major component of female homicide worldwide.^1^^,^^2^^,^^5^^,^^19^ These sources help explain why femicide must be assessed within a broader continuum of prior abuse, control, and institutional response.

The contribution of epidemiological evidence differs from the contribution of autopsy evidence. Epidemiological studies identify population-level risk factors, surveillance gaps, and prevention priorities.^1^, ^2^, ^3^, ^4^, ^5^, ^6^^,^^16^^,^^17^^,^^19^^,^^20^ Forensic studies document mechanisms of death, injury patterns, and medico-legal variables in individual cases.^7^, ^8^, ^9^, ^10^, ^11^^,^^18^ The two evidence layers should be integrated but not conflated.

### Legal classification and expert testimony

The forensic expert should not replace the court in assigning legal categories. However, expert testimony may assist legal classification by providing technically grounded statements about compatibility, reconstruction, limitations, and evidentiary support. Reports should distinguish observation from inference and should explicitly acknowledge what the autopsy can and cannot establish.^7^^,^^10^^,^^17^

Appropriate forensic phrasing may include: “The distribution and multiplicity of injuries are compatible with repeated close-range assault”; “The neck findings are compatible with manual strangulation, subject to correlation with scene and investigative data”; “The absence of defensive wounds does not exclude femicide”; and “The autopsy findings alone do not establish gender-related motivation”. This phrasing preserves scientific rigor and reduces the risk of anatomical determinism.

### Integrated medico-legal framework for femicide assessment

**Fig. 2:**
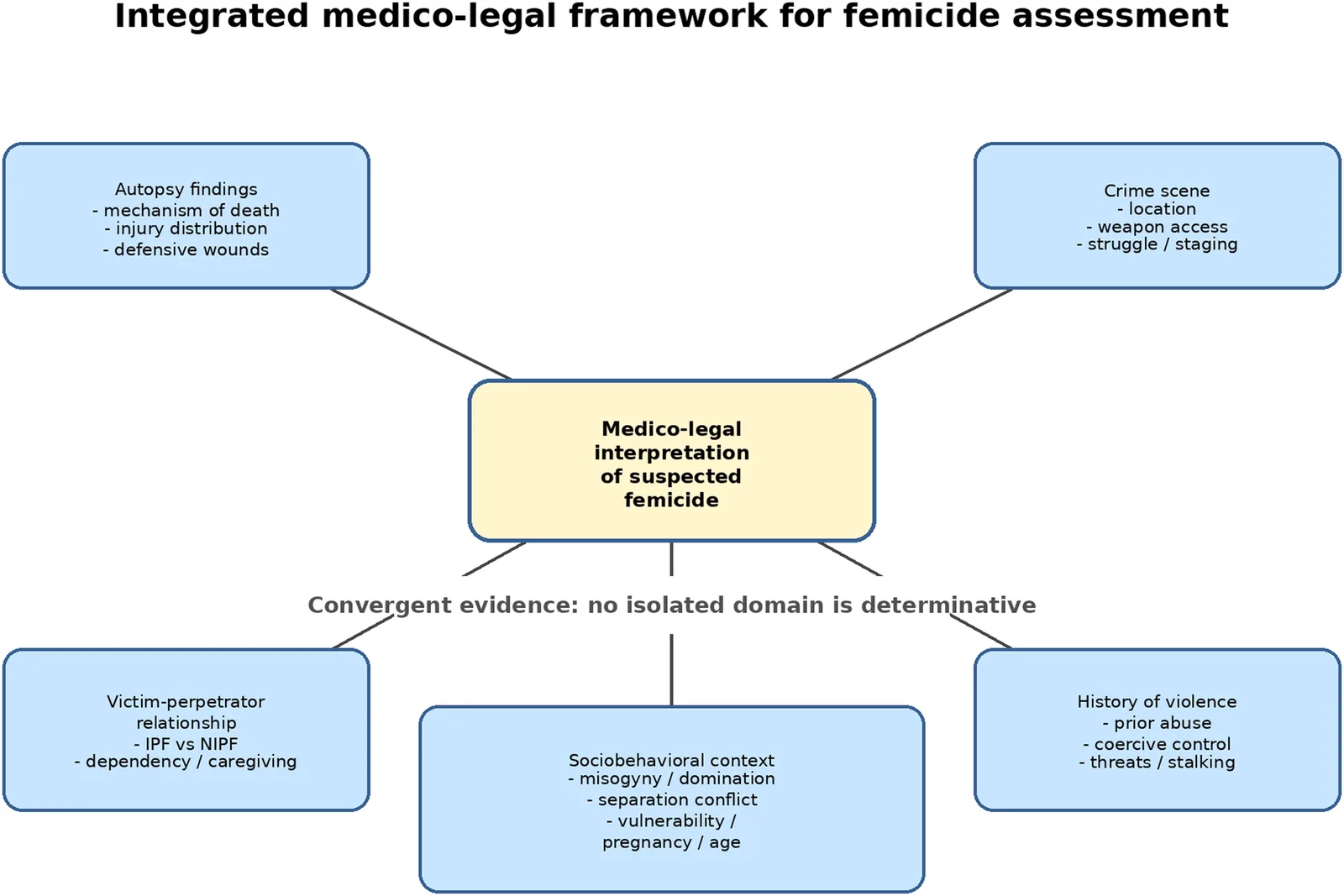
Integrated medico-legal framework for femicide assessment. The figure shows that autopsy findings, scene reconstruction, relationship data, prior violence, and sociobehavioral context interact to support proportionate medico-legal interpretation. No isolated domain is determinative.

To operationalize the interpretative approach proposed in this review, we retained and refined the integrated medico-legal framework for femicide assessment, presented in Fig. 2. This framework is not intended to function as a diagnostic algorithm or as a substitute for judicial classification. Rather, it provides a structured model for organizing evidentiary domains that should be considered when forensic findings are interpreted in suspected gender-related killings.

The framework is based on the interaction of five complementary domains: autopsy findings, crime scene investigation, victim-perpetrator relationship, prior history of violence, and sociobehavioral context. Autopsy findings include the mechanism of death, injury pattern, anatomical distribution of trauma, presence or absence of defensive wounds, evidence of asphyxia, sexual violence, or excessive violence. Crime scene investigation contributes information on location, weapon availability, body position, signs of struggle, concealment, staging, or domestic context. The victim-perpetrator relationship is essential for distinguishing intimate partner femicide from non-intimate partner femicide, but it cannot be inferred from morphology alone.^4^^,^^5^^,^^18^

The added value of the framework lies in explicitly showing that no single domain is sufficient by itself. For example, overkill or craniofacial injuries may support the hypothesis of close-contact or excessive violence, but they do not establish gender-related motivation unless interpreted together with relational and contextual evidence.^7^, ^8^, ^9^ Similarly, a single firearm wound may appear morphologically non-specific, but may still occur within a well-documented history of coercive control, threats, separation conflict, or prior intimate partner violence.^4^^,^^5^^,^^18^^,^^19^

Prior history of violence is a distinct evidentiary domain. This includes police reports, medical records, restraining orders, emergency department visits, witness statements, documented threats, stalking, coercive control, or prior episodes of strangulation. Such information is often unavailable to the forensic pathologist at the time of autopsy, but its absence should be recognized as an institutional limitation rather than as evidence against femicide.^2^, ^3^, ^4^, ^5^^,^^17^^,^^20^

Sociobehavioral context further strengthens interpretation by situating the death within broader patterns of gender-based violence, vulnerability, dependency, misogyny, sexual violence, separation conflict, jealousy, financial control, or caregiving asymmetry. This domain is particularly relevant in cases involving elderly women, socially isolated victims, pregnancy or postpartum status, disability, or prior exposure to intimate partner violence.^4^^,^^5^^,^^13^^,^^16^^,^^19^

Fig. 2 therefore reinforces the central thesis of this review: femicide assessment requires contextual integration rather than anatomical determinism. The forensic expert should document and interpret morphology with technical precision, but medico-legal characterization depends on convergence between anatomical, investigative, relational, and contextual evidence. This structured approach may improve consistency in forensic reporting, reduce underrecognition, and support transparent communication with legal authorities.

### Structural determinants, underreporting, and institutional fragmentation

Underrecognition of femicide can occur when forensic systems document only the immediate cause of death and do not capture gender-related context. Fragmentation between forensic institutes, police records, health services, courts, and social assistance systems may prevent identification of prior violence, protective orders, stalking, coercive control, or separation conflict.^2^^,^^3^^,^^17^^,^^20^

Actionable improvements include standardized autopsy templates for suspected gender-related killings; mandatory documentation of relationship data when available; structured fields for prior violence, protective orders, separation, stalking, pregnancy/postpartum status, sexual violence indicators, and elder vulnerability; training in non-fatal strangulation and coercive control; and interinstitutional linkage between forensic, police, judicial, social, and health data systems.^2^^,^^10^^,^^17^^,^^20^

### Limitations

This review has limitations. First, the number of primary forensic autopsy studies is limited, and several sources differ in case definitions, legal categories, sampling, and reporting quality. Second, the available literature is geographically uneven, with limited evidence from regions where femicide burdens may be high. Third, no formal quality assessment tool such as STROBE or QUADAS was applied because this was a structured narrative review rather than a systematic review or diagnostic accuracy study. Fourth, epidemiological studies were used for contextual framing and were not subjected to systematic synthesis.

These limitations mean that the conclusions should be interpreted as a critical medico-legal synthesis, not as evidence of diagnostic accuracy. The review does not identify a forensic pattern capable of diagnosing femicide independently. Instead, it supports an integrated framework in which anatomical findings contribute to, but do not determine, medico-legal classification.

### Synthesis

In summary, femicide is associated with recurrent but non-specific forensic patterns. Overkill, craniofacial trauma, strangulation, firearm injuries, and defensive wounds may contribute to reconstruction, but none is sufficient to establish gender-related motivation in isolation.^7^, ^8^, ^9^, ^10^, ^11^^,^^18^ The distinction between intimate partner femicide and non-intimate partner femicide depends primarily on relational, contextual, and investigative evidence rather than on morphology alone.^4^^,^^5^^,^^18^

The most defensible medico-legal approach is therefore integrative, structured, and cautious. Autopsy findings should be interpreted together with crime scene information, victim-perpetrator relationship, prior history of violence, sociobehavioral context, and institutional records. This approach avoids anatomical determinism, improves forensic transparency, supports judicial decision-making, and contributes to more accurate surveillance of gender-related killings.^2^^,^^3^^,^^17^^,^^20^

### Take-home message

The main implication of this review is that femicide should not be approached as a purely morphological diagnosis. Autopsy findings are indispensable for documenting mechanism of death, injury distribution, excessive violence, defensive wounds, and signs of asphyxia or sexual violence; however, none of these findings is specific enough to establish gender-related motivation when interpreted in isolation. The most robust medico-legal assessment requires convergence between forensic morphology, crime scene findings, victim-perpetrator relationship, previous history of violence, and sociobehavioral context. Therefore, forensic experts should avoid categorical legal conclusions based solely on injury patterns and should instead provide structured, evidence-based interpretations that support judicial classification while preserving scientific caution.

## Conclusion

Femicide presents recurrent forensic features, including overkill, craniofacial and cervical trauma, asphyxial mechanisms, defensive wounds, and weapon-specific injury patterns. These features are important for reconstruction and may support suspicion of gender-related violence when interpreted in context. However, they are not specific and do not allow reliable differentiation between intimate partner femicide and non-intimate partner femicide when considered in isolation.

The medico-legal classification of femicide should therefore be based on an integrated evidentiary model. Autopsy findings must be interpreted together with scene reconstruction, victim-perpetrator relationship, prior violence, coercive control, weapon access, and sociobehavioral context. Standardized documentation, structured reporting, and interdisciplinary data integration may improve forensic consistency, legal communication, and public health surveillance.

## Authors’ contributions

Conceptualization: Author 1 and Author 2. Methodology: Author 1 and Author 2. Literature review and data extraction: Author 1 and Author 2. Formal analysis and interpretation: Author 1 and Author 2. Writing-original draft: Author 1. Writing-review and editing: Author 1 and Author 2. Supervision: Author 2. Both authors approved the final version of the manuscript.

## AI use disclosure

The authors used generative artificial intelligence tools to assist in language editing, text refinement, and organization of the manuscript. All outputs were critically reviewed, validated, and substantially revised by the authors, who take full responsibility for the content, accuracy, and integrity of the work. No artificial intelligence tools were used for data collection, data analysis, or generation of original scientific results.

## Use of artificial intelligence

During manuscript revision, generative artificial intelligence was used only as an editorial support tool for language refinement, structural organization, and formatting. The authors reviewed, verified, and approved all content, references, interpretations, and conclusions, and remain fully responsible for the integrity of the manuscript.

### Ethics approval

Ethics committee approval was not required because this study is a narrative review based exclusively on previously published literature and did not involve human participants, identifiable personal data, biological samples, medical records, clinical images, or direct patient interaction.

### Informed consent

Informed consent was not applicable because no patients, volunteers, organ or tissue donors, identifiable personal information, clinical images, or individual case details were included in this narrative review.

## Declaration of competing interest

The authors declare no conflicts of interest.

## Data Availability

All data analyzed in this narrative review are derived from previously published studies cited in the manuscript. No new datasets were generated or analyzed during the present study.
